# Longitudinal Patterns of Systolic Blood Pressure, Diastolic Blood Pressure, Cardiorespiratory Fitness, and Their Association With Dementia Risk: The HUNT Study

**DOI:** 10.1093/gerona/glae161

**Published:** 2024-06-19

**Authors:** Maren Lerfald, Heather Allore, Tom I L Nilsen, Rannveig S Eldholm, Nicolas Martinez-Velilla, Geir Selbæk, Linda Ernstsen

**Affiliations:** Faculty of Medicine and Health Science, Department of Public Health and Nursing, Norwegian University of Science and Technology, Trondheim, Norway; Clinic of Medicine, St. Olavs Hospital, Trondheim University Hospital, Trondheim, Norway; Department of Internal Medicine, Yale School of Medicine, Yale University, New Haven, Connecticut, USA; Department of Biostatistics, Yale School of Public Health, Yale University, New Haven, Connecticut, USA; Faculty of Medicine and Health Science, Department of Public Health and Nursing, Norwegian University of Science and Technology, Trondheim, Norway; Clinic of Anaesthesia and Intensive Care, St. Olavs Hospital, Trondheim University Hospital, Trondheim, Norway; Department of Geriatrics, Clinic of Medicine, St. Olavs Hospital, Trondheim University Hospital, Trondheim, Norway; Faculty of Medicine and Health Science, Department of Neuromedicine and Movement Science, Norwegian University of Science and Technology, Trondheim, Norway; Navarrabiomed, Hospital Universitario de Navarra, UPNA, IdiSNA, Pamplona, Spain; Norwegian National Centre for Ageing and Health, Vestfold Hospital Trust, Oslo, Norway; Department of Geriatric Medicine, Oslo University Hospital, Oslo, Norway; Faculty of Medicine and Health Science, Department of Public Health and Nursing, Norwegian University of Science and Technology, Trondheim, Norway; Clinic of Medicine, St. Olavs Hospital, Trondheim University Hospital, Trondheim, Norway

**Keywords:** Cardiovascular, Cognition, Prevention

## Abstract

**Background:**

High blood pressure and poor cardiorespiratory fitness are independent risk factors for dementia. However, few studies have examined if combined longitudinal patterns of these modifiable risk factors are associated with dementia risk.

**Methods:**

In this prospective cohort study, we used data from the population-based Trøndelag Health (HUNT) Study, Norway. We applied group-based multidimensional trajectory modeling to identify age-specific multidimensional trajectories of SBP, DBP, and estimated cardiorespiratory fitness across 3 surveys (HUNT1, 1984–1986 to HUNT3, 2006–2008). Dementia was diagnosed in the HUNT4 70+ substudy in 2017–2019. We used multivariate logistic regression to estimate odds ratios (ORs) and risk differences (RDs) of dementia.

**Results:**

In total, 7 594 participants (54.9% women) were included, with a mean age of 44.7 (*SD* 6.3) years at HUNT1. Dementia was diagnosed in 1 062 (14.0%) participants. We identified 2 multidimensional trajectories throughout adulthood within 3 age groups: one with higher systolic blood pressure (SBP) and diastolic blood pressure (DBP), and lower estimated cardiorespiratory fitness (the poorer group), and one with lower SBP and DBP, and higher cardiorespiratory fitness (the better group). After adjustment for sex, apolipoprotein E ε4 status, education, marital status, and diabetes, the better group had consistently lower risk of dementia in all age groups with the lowest OR in the middle-aged group of 0.63 (95% confidence intervals [95% CI]: 0.51, 0.78) with corresponding RD of −0.07 (95% CI: −0.10, −0.04).

**Conclusions:**

Having a beneficial multidimensional trajectory of SBP, DBP, and cardiorespiratory fitness in adulthood was associated with reduced dementia risk. Aiming for optimal SBP, DBP, and estimated cardiorespiratory fitness throughout adulthood may reduce dementia risk.

Dementia is a universal term covering several diseases affecting cognitive function and activities of daily living (1). In 2019, the global prevalence of dementia was 57 million, and by 2050 this number is estimated to increase to 153 million (2). In Norway, this number is expected to reach 237 thousand people by 2050, compared with 101 thousand people in 2020 (3). Dementia has currently no cure, and therefore prevention has gained increasing attention. Normotensive blood pressure (BP) (4) and higher levels of cardiorespiratory fitness have both been found to reduce dementia risk (5,6). A recent report estimated 12 modifiable risk factors to account for about 40% of dementias around the world, and aiming for systolic BP (SBP) < 130 mm Hg in midlife and sustaining physical activity in mid- and possibly late-life were some of the recommended strategies to reduce dementia risk (4).

The association between BP and dementia has been shown to be dependent on age and whether SBP or diastolic BP (DBP) are high or low (7). Increased BP in midlife has been associated with increased dementia risk, while both high and low BP in late-life have been associated with increased dementia risk (7–10). However, the strongest relation appears to be in midlife, and midlife hypertension has been found to be associated with increased risk of dementia (7). There is an ongoing discussion of the optimal BP level for lowering dementia risk, where the SPRINT MIND Study found intensive BP control to reduce the risk of combined mild cognitive impairment or probable dementia (11), while others did not find evidence for lower BP targets in prevention of dementia and cognitive decline (12). This topic needs further investigations.

Although physical activity represents any bodily movement that requires energy expenditure, cardiorespiratory fitness represents the capacity of the cardiovascular and respiratory systems to transport oxygen to skeletal muscle and vital organs (13). Cardiorespiratory fitness is mainly improved through regular aerobic physical activity of sufficient intensity (14). Both physical activity (15) and cardiorespiratory fitness (6,16) have been associated with reduced dementia risk. As equipment to measure cardiorespiratory fitness is not routinely available in clinical practice, algorithms have been developed and provide reasonably accurate estimates (13). There are fewer studies investigating cardiorespiratory fitness patterns over time; however, it has been shown that different patterns of change are associated with different dementia risk, and that improving or maintaining high cardiorespiratory fitness relative to age decreases the risk for dementia (17).

There is a lack of research on combinations of risk factors, interactions of risk factors, the role of sex, life-course exposures, and studies that consider different age groups (18). Studies investigating an outcome with a modifiable risk factor as exposure are prone to bias due to reverse causation (19). To handle this potential bias, studies with long follow-up time are needed. Therefore, our aim is to identify multidimensional trajectories of SBP, DBP, and estimated cardiorespiratory fitness over 24 years in mid- to late-life, stratified by age groups, and investigate how these trajectories are associated with dementia assessed approximately 10 years after the last BP measure and cardiorespiratory fitness estimation. This results in an overall follow-up time of more than 30 years. As it has been noted that an association of BP and dementia might be different according to sex (20), and that there might be different risk factors across the life span among men and women (21); we will investigate if there is an interaction between sex and the multidimensional trajectories.

## Method

### Study Sample

In this prospective cohort study, all data were obtained from the Norwegian population-based Trøndelag Health Study (HUNT). The adult population of people 20 years and older, in the entire county of North Trøndelag, had been invited to take part in questionnaires, interviews, clinical examinations, laboratory measurements, and to give biological samples in 4 separate and following surveys: HUNT1 (1984–1986), HUNT2 (1995–1997), HUNT3 (2006–2008), and HUNT4 (2017–2019) (22). At HUNT1, 89.4% participated, 69.5% participated at HUNT2, 54.1% and 54.0% at HUNT3 and HUNT4, respectively. Details about the HUNT Study are reported elsewhere (22,23).

**Figure 1.**
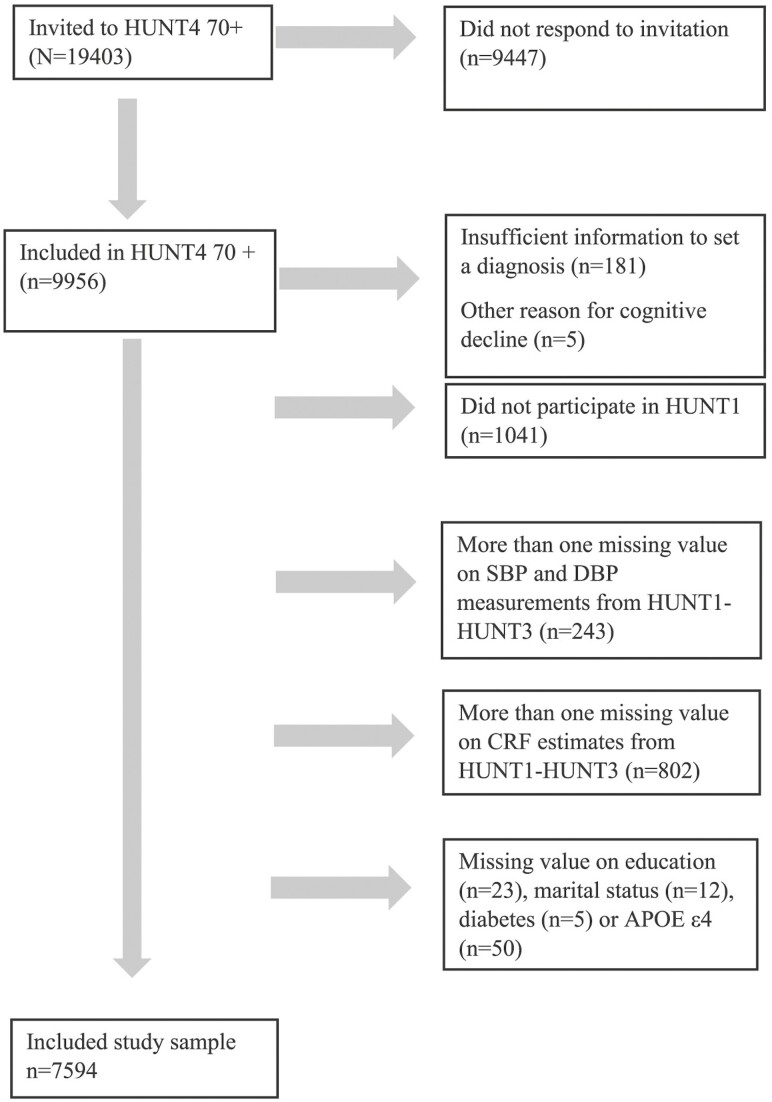
Flowchart of included study sample.

At HUNT4, people aged 70 years and older were invited to the substudy HUNT4 70+, which included a clinical cognitive assessment (3). In this study, 19 403 individuals were invited, and we included those who participated in HUNT4 70+ (*n* = 9 956, 51.3%). We excluded those who had insufficient information from the cognitive assessment (*n* = 181), other reasons for cognitive decline (*n* = 5), or did not participate in HUNT1 (*n* = 1 041). In addition, we excluded those who had <2 valid systolic and DBP measures (*n* = 243) or had <2 measures of estimated cardiorespiratory fitness from HUNT1 to HUNT3 (*n* = 802), and we excluded those who had missing information on education (*n* = 23), marital status (*n* = 12), diabetes (*n* = 5), or apolipoprotein E ε4 (APOE ε4; *n* = 50). Finally, our study sample included 7 594 participants (Figure 1). As we expected BP and cardiorespiratory fitness to differ by age, participants were grouped based on their age in years at HUNT1: youngest age group: >35 and ≤45 years (*n* = 4 613); middle-aged groups: >45 and ≤55 years (*n* = 2 347); oldest-aged group: >55 years (*n* = 634).

### Dementia Assessment

The cognitive assessment in the HUNT4 70+ substudy included test of cognitive function, evaluation of neuropsychiatric symptoms, symptom development, daily life functioning, subjective cognitive decline, and interviews with next-of-kin (3). Based on the retrieved information, 2 medical doctors from a group of 9, with expertise in either neurology, old-age psychiatry, or geriatrics independently made a diagnosis based on the DSM-5 diagnostic criteria (24). If their conclusions differed, a third specialist was consulted. For the present study, participants were categorized into living with all-cause dementia or no dementia. Details on the cognitive assessment at HUNT4 70+ can be found in Gjøra et al. (3).

### Systolic and Diastolic Blood Pressure

We used clinically measured SBP and DBP levels from HUNT1, HUNT2, and HUNT3 (25). At HUNT1, SBP and DBP were measured 2 times by technicians or nurses using a mercury sphygmomanometer following a standardized protocol. At HUNT2 and HUNT3, SBP and DBP were measured 3 times following standardized protocol, and oscillometry-based devices were used. In this study, the second measure at HUNT1 and the mean of the second and third measure at HUNT2 and HUNT3 were used in the analysis.

### Estimated Cardiorespiratory Fitness

Validated sex-specific algorithms were used to estimate cardiorespiratory fitness at HUNT1, HUNT2, and HUNT3, and the information needed in the algorithms was retrieved from the corresponding surveys. At HUNT1 and HUNT3, the same algorithm was used and included age, body mass index (BMI), resting heart rate (RHR), and a physical activity index (PA-I) (26). BMI and RHR were objectively and standardly measured (23), while the PA-I is based on the previously validated physical activity questionnaire assessing frequency, duration, and intensity (27,28). At HUNT2, a different validated questionnaire was used to assess physical activity (29). Therefore, a different sex-specific algorithm was used (30). This algorithm included clinically measured waist circumference (WC) and RHR, self-reported physical activity, and age. The physical activity measure in this algorithm had 2 possible values (0, 1) corresponding to meeting or not meeting the current physical activity recommendations (30,31). Finally, for comparability, cardiorespiratory fitness was standardized by sex in the defined age groups such that there was a mean of zero and a standard deviation of 1 (32), where a higher score indicates higher cardiorespiratory fitness.

Cardiorespiratory fitness algorithm at HUNT1 and HUNT3:

Women: 70.77 − (0.244*Age) − (0.749*BMI) − (0.107*RHR) + (0.213*PA-I)

Men: 92.05 − (0.327*Age) − (0.933*BMI) − (0.167*RHR) + (0.257*PA-I)

Cardiorespiratory fitness algorithm at HUNT2:

Women: 78.00 − (0.297*Age) − (0.270*WC) − 0.110*RHR + (2.674*PA)

Men: 105.91 − (0.334*Age) − (0.402*WC) − (0.144*RHR) + (3.102*PA)

### Covariates

We controlled for possible confounding factors at HUNT1 and included the variables sex (men, women(ref)), education (primary(ref)), high school, college or university ≤4 years, college or university >4 years), marital status (married or not(ref)), diabetes (yes, no(ref)), APOE ε4 (absent(ref), 1 or 2 alleles present), smoking (never(ref), former, daily smoker), alcohol frequency last 14 days (abstainer(ref), did not drink, 1–4 times, more than 5 times) and anxiety- and depression-index (ADI-4) (33) (*Z*-score). If there was missing information on education at HUNT1, we included information from HUNT2. All covariates are self-reported except information on APOE ε4 (34).

### Statistical Analyses

Characteristics were calculated with means and standard deviations for continuous variables and with frequencies and percentages for the categorical variables. We used an extended version of the group-based trajectory modeling called group-based multidimensional trajectory modeling to assess longitudinal patterns of SBP, DBP, and estimated cardiorespiratory fitness stratified by age groups (35,36). First, we identified separate trajectories for each of the 3 trajectory outcomes assessing model fit criteria including (a) Bayesian information criterion; (b) average posterior probability; (c) odds of correct classification; (d) required group size estimated >5.0% of sample; and (e) proportion of participants whose highest probability of group membership≤ 0.7. For each individual trajectory outcome, we investigated different number of groups and whether the best fit was linear or quadratic for the above criteria. Based on the model fit from the individual trajectory modeling, we estimated the multidimensional trajectories using the best order (ie, linear or quadratic) for each outcome. We compared the optimal number of multidimensional trajectory groups using the same criteria. Each person was assigned the trajectory for which they had the highest probability of membership. We used the traj package in Stata (37).

**Figure 2.**
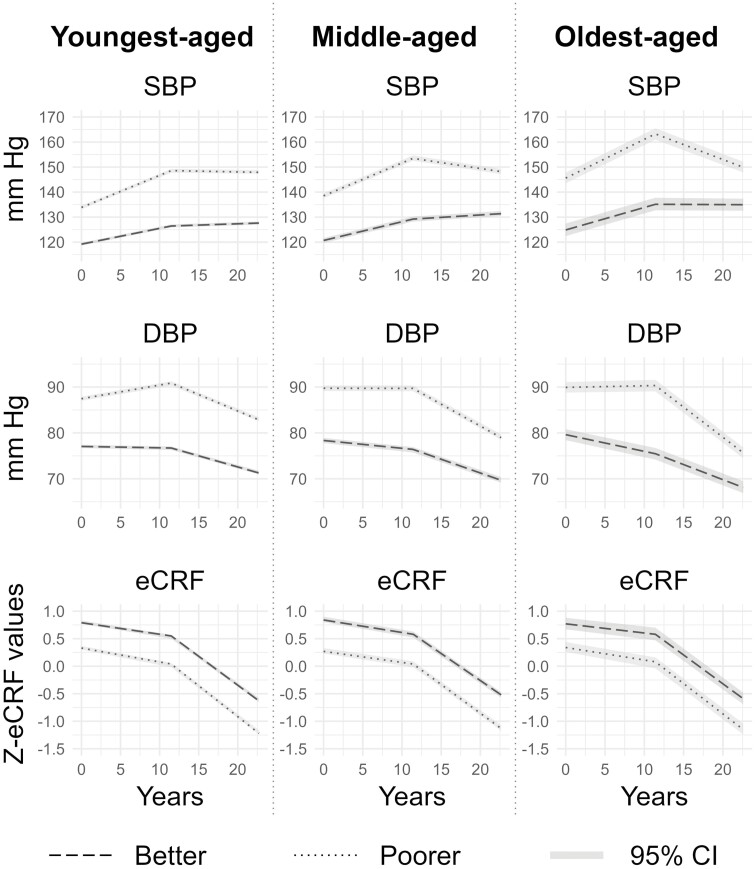
Multidimensional trajectories (read as columns) of systolic blood pressure (SBP), diastolic blood pressure (DBP), and estimated cardiorespiratory fitness (eCRF) stratified by age groups. The multidimensional trajectories are based on three repeated measures from HUNT1 (baseline = 0 years), HUNT2, and HUNT3. *Note*: *Z-eCRF* = Z-transformed eCRF values.

To investigate the association between the multidimensional trajectories and dementia, we used logistic regression with the multidimensional trajectory groups as the independent variable and dementia as the dependent variable stratified by age groups. Our first model was unadjusted, and our second model was adjusted for sex, education, marital status, diabetes, and APOE ε4. To assess if sexes differed by the multidimensional trajectory groups, we included an interaction term in our fully adjusted model. We report relative and absolute estimates including odds ratios (ORs) and risk differences (RDs) with 95% confidence intervals (95% CI) (38). We set a significance level of *p* value ≤ .05. We used Stata MP version 18.0 (StataCorp. 2023. *Stata Statistical Software: Release 18*. StataCorp LLC, College Station, TX) for all analyses. Figure 2 was made using R Statistical Software version 4.3.2 (2023-10-31 ucrt) (R Foundation for Statistical Computing, Vienna, Austria).

### Sensitivity Analysis

The primary analysis was a complete case analysis including in total *n* = 7 594, stratified by age groups. We performed sensitivity analyses to investigate the robustness of the results. First, we estimated the *E*-value for the point estimates and confidence intervals for all regression models, which indicates the strength an unmeasured confounder would need to have with the exposure and the outcome to take away a potential association (39,40). Second, we included the covariates smoking, alcohol frequency, and ADI-4 on a reduced data set (overall total *n* = 6 673 because of missing data) and reran a third model of the logistic regressions.

### Ethics

In all surveys of the HUNT Study, participants provided informed written consent. This study was approved by the Regional Committee of Medical and Health Research Ethics in Norway (REK sør-øst D 2017/382). Use and storage of data follow the General Data Protection Regulation.

### Data Availability

Data can be obtained from a third party (from the HUNT database, url: HUNT Databank (ntnu.no)) and are not available for the public.

## Results

In total, 7 594 (mean age (*SD*) 44.7 (6.3) 54.9% women at HUNT1) individuals were included. Dementia was diagnosed in 14.0% of the study sample at HUNT4 70+. In the youngest age group dementia was diagnosed in 6.4% of the sample, in the middle-aged and the oldest-aged groups dementia was diagnosed in 19.8% and 47.8% of the sample, respectively. Descriptives of the study samples are presented in Table 1.

**Table 1.** Descriptives at Baseline (HUNT1)

	Total Study Sample, *n* = 7 594	Youngest-Aged Group: >35 and ≤45 y, *n* = 4 613	Middle-Aged Group: >45 and ≤55 y, *n* = 2 347	Oldest-Aged Group: >55 y, *n* = 634	Total, *n*
	Mean (*SD*) or *n* (%)				
Age	44.65 (6.34)	40.42 (2.41)	49.19 (2.79)	58.61 (2.94)	7 594
Sex					
Women	4 167 (54.87)	2 423 (52.53)	1 357 (57.82)	387 (61.04)	7 594
Marital status					
Married	6 853 (90.24)	4 169 (90.38)	2 147 (91.48)	537 (84.70)	7 594
SBP (mm Hg)	127.38 (15.33)	125.23 (14.05)	129.33 (15.88)	135.73 (18.15)	7 565
DBP (mm Hg)	82.43 (9.97)	81.33 (9.74)	83.88 (10.18)	85.02 (9.68)	7 563
Estimated CRF (mL/kg)	38.56 (6.44) [12.81, 63.69]	40.30 (6.01) [12.81, 63.69]	36.75 (6.09) [16.18, 59.39]	33.08 (5.59) [15.82, 48.36]	6 905
Education					7 594
Primary	3 475 (45.76)	1 802 (39.06)	1 255 (53.47)	418 (65.93)	
High school	2 732 (35.98)	1 822 (39.50)	746 (31.79)	164 (25.87)	
College/Uni ≤4 y	776 (10.22)	568 (12.31)	180 (7.67)	28 (4.42)	
College/Uni >4 y	611 (8.05)	421 (9.13)	166 (7.07)	24 (3.79)	
Smoking					6 969
Never	3 220 (46.20)	1 782 (42.80)	1 100 (50.07)	338 (55.59)	
Former	1 999 (28.68)	1 222 (29.35)	599 (27.26)	178 (29.28)	
Daily	1 750 (25.11)	1 160 (27.86)	498 (22.67)	92 (15.13)	
Alcohol use frequency last 14 d					6 926
Abstainer	578 (8.35)	257 (6.19)	231 (10.61)	90 (15.08)	
Did not drink	2 853 (41.19)	1 558 (37.52)	986 (45.29)	309 (51.76)	
1–4 times	3 075 (44.40)	2 075 (49.98)	821 (37.71)	179 (29.98)	
≥5 times	420 (6.06)	262 (6.31)	139 (6.38)	19 (3.18)	
ADI^*^	−0.00 (3.06)	−0.04 (3.06)	0.00 (3.03)	0.21 (3.23)	6 741
Diabetes	39 (0.51)	22 (0.48)	11 (0.47)	6 (0.95)	7 594
APOE ε4	2 267 (29.85)	1 437 (31.15)	658 (28.04)	172 (27.13)	7 594

*Notes:* ADI = anxiety- and depression-index; APOE ε4 = apolipoprotein E ε4; CRF = cardiorespiratory fitness; DBP = diastolic blood pressure; SBP = systolic blood pressure; Uni = university.

^*^ *Z*-score.

### Multidimensional Trajectories

The individual trajectories of SBP, DBP, and estimated cardiorespiratory fitness showed the best overall fit for 2 and 3 groups. Based on this information, we compared multidimensional trajectories between the 2 and 3 groups. In all age groups, the best multidimensional fit was found for 2 groups. Thus, the 2-group model was found to be the overall best choice for our analysis (Supplementary Table 1, additional figures available upon request). Among the different age groups, the SBP trajectory in the better trajectory group was similar starting at a normotensive level and with a slight increase to flatten over time. In the poorer trajectory group, the SBP started higher and had a greater increase in the start of the trajectories and flattened to decrease in the end. This was particularly notable in the oldest-aged group. For consistency throughout the article, the 2 multidimensional trajectories will be referred to as “better” and “poorer.” Among the youngest-aged group, the intercept for the better SBP trajectory was 119.17 mm Hg and for the poorer SBP trajectory group, 133.87 mm Hg. Among the middle-aged group, the corresponding numbers were 120.66 and 138.50, and 124.85 and 145.65 for the oldest-aged group. The DBP trajectories varied according to the intercept and were stably decreased over time in both multidimensional trajectory groups and across age groups. In the better trajectory group, the DBP intercept ranged from 77.05 to 79.62 mm Hg across age groups, and in the poorer trajectory group, the DBP intercept ranged from 87.46 to 89.94 mm Hg across age groups. Similar to the DBP, the estimated cardiorespiratory fitness trajectory varied according to the intercept and decreased similarly in the 2 trajectories over time and across the age groups (Figure 2). All parameter estimates for the multidimensional trajectories are presented in Supplementary Table 2.

### Regression Analysis

We chose the hypothesized poorer trajectory group (higher SBP and DBP and lower cardiorespiratory fitness) as the reference group for the regression analysis in all age strata. The results from the logistic regression analyses and *E*-values are presented in Table 2.

**Table 2.** Odds Ratios and Risk Differences for Dementia by Age Groups. Estimates Are Presented for the Better Multidimensional Trajectory Compared to the Poorer. *E*-Values Are Presented for Each Regression Model

Age Groups		*N*	Odds Ratio (95% CI)	Absolute Risk Difference (95% CI)	*E*-Value Point Estimate (*E*-Value CI)
>35 and ≤45	Model 1^*^	4 613	0.67 (0.53, 0.84)	−0.02 (−0.04, −0.01)	2.37 (1.65)
	Model 2^**^	4 613	0.72 (0.56, 0.91)	−0.02 (−0.03, −0.01)	2.14 (1.42)
>45 and ≤55	Model 1^*^	2 347	0.60 (0.49, 0.74)	−0.08 (−0.11, −0.05)	1.89 (1.59)
	Model 2^**^	2 347	0.63 (0.51, 0.78)	−0.07 (−0.10, −0.04)	1.83 (1.52)
>55	Model 1^*^	634	0.62 (0.45, 0.85)	−0.12 (−0.20, −0.04)	1.86 (1.39)
	Model 2^**^	634	0.66 (0.48, 0.91)	−0.10 (−0.18, −0.02)	1.76 (1.27)

*Notes:* CI = confidence interval.

^*^Unadjusted.

^**^Adjusted for sex, APOE ε4 status, education, marital status, and diabetes.

We found the adjusted OR and adjusted RD in all age strata to be protective for the multidimensional trajectories starting with SBP and DBP at a normotensive level and cardiorespiratory fitness at higher level. In the youngest age group, OR = 0.72 (95% CI: 0.56, 0.91) and RD = −0.02 (95% CI: −0.03, −0.01), in the middle-aged group, OR = 0.63 (95% CI: 0.51, 0.78) and RD = −0.07 (95% CI: −0.10, −0.04), and in the oldest-aged group, OR = 0.66 (95% CI: 0.48, 0.91) and RD = −0.10 (95% CI: −0.18, −0.02). The results are significant in all age groups and appear to be strongest in the middle-aged group. We further stratified all 3 age groups by sex and reran the logistic regression (Supplementary Table 3). The differences according to sex were notable in the youngest- and oldest-aged groups, and particularly in the oldest-aged group, where the OR among men was 0.46 (95% CI: 0.26, 0.79) and the OR among women was 0.83 (0.55, 1.25). However, the statistical interaction between the multidimensional trajectories and sex was nonsignificant in all age strata. All *E*-value point estimates were >1.75, and including smoking status, alcohol use frequency, and the ADI-4 in the regression models had similar results as our main analysis (Supplementary Table 4).

## Discussion

Our study contributes to increased knowledge on combinations of risk factors in mid- to late-life and their relation to dementia in older age. We identified 2 distinct multidimensional trajectories of SBP, DBP, and estimated cardiorespiratory fitness across 3 mid- to late-life age groups. Those assigned to the multidimensional trajectory where BP started at a normotensive level and cardiorespiratory fitness at higher level regardless of age were found to have lower adjusted OR and adjusted RD for dementia compared to those with poorer trajectories. We did not find support for any statistical interaction between sex and the multidimensional trajectories; however, we observed differences in OR between women and men in the youngest, and especially in the oldest-aged group.

We modeled multidimensional trajectories of SBP, DBP, and estimated cardiorespiratory fitness over 24 years and their association with future dementia. Our findings are in line with other research investigating BP and dementia (41), cardiorespiratory fitness and dementia (17), and combined physical activity and SBP and dementia (42). A previous study investigating vascular risk factors including BP, at ages 55, 65, 70, 75, and 80 and a 10-year risk of incident dementia 10 years after the exposure measure, found SBP to be among the most important vascular risk factors at age of 55, but not later (43). This supports our finding that the middle-aged group had a slightly stronger association. However, they did not investigate the patterns of BP development.

The SBP among the poorer multidimensional trajectories is starting at a level >130 mm Hg in all age groups and >140 mm Hg in the oldest-aged group. The threshold of 130 mm Hg in SBP in midlife (at the age of 50) for increased dementia risk is supported by prospective findings from the Whitehall II cohort study (44). Interestingly, in our study, the SBP across the poorer multidimensional trajectory group, particularly in the oldest-aged group, increased more between HUNT1 and HUNT2 and decreased between HUNT2 and HUNT3. Similar patterns have been observed in previous research among people with dementia (8). Because we only have one assessment of dementia, we do not know when it occurred. Therefore, we cannot guarantee that our findings are free from reverse causation, especially considering that preclinical stages could contribute to reverse causality as well. This would be of particular interest in the oldest-aged group. However, with 10 years between last exposure measure and the dementia assessment, we feel confident that this potential bias would not be strong enough to affect our results notably (45). In addition, it has been suggested that a decrease in BP could be related to closeness to death rather than related to age or dementia (46), which could be an explanation for the more prominent pattern in the oldest age group. However, in the HUNT Study, an overall decrease in BP especially between HUNT2 and HUNT3 was found and the decrease increased with age, and this could not be explained by increase in use of BP medications alone (25), which could be an additional explanation for the pattern in this case. It should be noted that there were some differences in the procedures and instruments used in HUNT1 compared to HUNT2-3. It has been shown that the results from the Dinamap device are slightly lower than those measured with a sphygmomanometer, especially for DBP (47). However, it is not likely that this would affect the results notably.

The estimated cardiorespiratory fitness trajectories were similar and seemed to differ mainly by the intercept both between age groups and trajectory groups. This indicates that having a higher cardiorespiratory fitness in midlife, and maintaining it was beneficial for dementia prevention in combination with a normotensive BP. This is in line with other studies investigating the association between cardiorespiratory fitness and dementia (16,17). The pattern of the cardiorespiratory fitness was primarily stable over time, and the intercept seemed to be most important. This might indicate that the trajectories mainly display a physiologically expected decrease by age, and it is less likely that persons change between these groups and that smaller changes in health behavior are not captured, due to the strong effect of age over time. Bearing in mind that physical activity is the greatest modifiable factor of cardiorespiratory fitness, it might display small changes in PA behavior in theses age groups. Stability rather than transition has been found to be more common among adults, and illustrates that adults fall into health behavior profiles quite early in adulthood (48). As all elements included in the cardiorespiratory fitness algorithm (BMI/WC, RHR, and PA) except age, are potentially modifiable and related to health behavior, the trajectories might display a stability in health behavior among the majority. This means that aiming to achieve a high cardiorespiratory fitness in earlier life, will probably be carried forward during midlife and later life, and benefit future dementia risk in combination with normotensive BP.

We did not find any statistical interaction between the multidimensional trajectories and sex in our study sample. Still, our findings support sex differences based on the stratified analysis. Ideally, the multidimensional trajectories should have been stratified according to sex in addition to age, but to maintain statistical power this was not done. A systematic review investigating a gender-modifying effect on the association between BP and dementia found that several of the studies indicated that higher SBP in midlife was associated with greater risk of dementia in women compared to men (49). However, they noted that studies rarely presented gender effects, and that the interpretation of the findings was difficult. This together indicates that the role of sex and gender should be further investigated in future research, where even bigger cohorts are investigated.

## Strengths and Limitations

This study has several strengths. Our study sample included 7 594 people and we had at least 2 objectively measured BP levels, and 2 estimated cardiorespiratory fitness values over 24 years in mid- to late-life, and dementia was assessed about 10 years later. We adjusted for several possible confounding factors at HUNT1.

Several limitations should be noted. The cardiorespiratory fitness variable was estimated and should ideally be objectively measured, and due to a different physical activity questionnaire at HUNT2 we had to use 2 different algorithms. However, the algorithms have been shown to be comparable to other published nonexercise models according to the accuracy of the models (28,30) and are reasonable alternatives when investigating large cohorts. In addition, we standardized the variable to maintain statistical strength, which in this case limits the information it holds. As we had no individual information about ethnicity, but the population in northern parts of Trøndelag are ethnically homogenous, our findings are mainly generalizable to a population of European ancestry (22). We should note that our study is particularly prone to survival bias when including people ≥70 years, meaning that our study sample is possibly healthier than the general population, which again could affect the generalizability. This especially counts for the oldest-aged group. Competing risk, particularly in relation to death, could affect both the observed multidimensional trajectories and the observed association between the multidimensional trajectories and dementia. This means that the multidimensional trajectories could look a bit different if survival bias was removed. For the observed association between the multidimensional trajectories and dementia, survival bias would make the association weaker. In fact, a recent prospective study identifying 3 distinct cardiovascular risk factor groups only found the group with vascular risk factors to be associated with incident Alzheimer's disease (AD), further, that the vascular-metabolic group had a significantly younger age of death compared with the reference group (84.3 vs 88.7 years) (50). Thus, the authors in the latter study suggested that selective mortality may contribute to the attenuated association between certain cardiovascular disease risk profiles and incident AD. In addition, we should note that the OR is comparable to the relative risk when the outcome is rare, and when the outcome is less rare, the OR will overestimate the risk. This should be kept in mind if interpreting the results from the 2 oldest groups as a measure of relative risk.

We controlled for covariates at baseline and found all *E*-value point estimates to be >1.75, indicating the strength an unmeasured confounder would need to have with the multidimensional trajectories and dementia to take away the association that we detected in our analysis. In addition, we included a sensitivity analysis where the covariates smoking, alcohol use frequency, and ADI-4 were included, which gave similar results as our main analysis. However, some of these covariates are time-varying and other confounding factors should ideally be considered, for example, dietary habits and prescribed medications, but this information was lacking. In this study, we did not investigate subtypes of dementia to maintain statistical power in our analysis, and we did not investigate the role of BP-lowering medication. This should be investigated further in future research.

In summary, having a beneficial multidimensional trajectory of SBP, DBP, and estimated cardiorespiratory fitness might reduce dementia risk in adults irrespective of age group. The association appears strongest for those in the middle-aged group being >45 and ≤55 years at baseline. Aiming for normotensive SBP, DBP, and a higher level of estimated cardiorespiratory fitness, in all age groups can be of high importance for dementia prevention at a public health level.

## Supplementary Material

glae161_suppl_Supplementary_Tables_S1-S4
