# Supplementary material for: Longitudinal Patterns of Systolic Blood Pressure, Diastolic Blood Pressure, Cardiorespiratory Fitness, and Their Association With Dementia Risk: The HUNT Study
Source: J Gerontol A Biol Sci Med Sci. 2024 Jun 19;79(8):glae161. doi: 10.1093/gerona/glae161 (PMC11266981; doi:10.1093/gerona/glae161)
Supplement: glae161_suppl_Supplementary_Tables_S1-S4 [file glae161_suppl_supplementary_tables_s1-s4.docx]

**Supplementary material**

**eTable 1** Model fit of the multidimensional trajectories comparing two and three groups.

**eTable 2** Parameter estimates for the two multidimensional trajectories.

**eTable 3** Adjusted oddsratio (aOR) with 95% confidence interval (95% CI) for dementia stratified by age group and sex, where the better multidimensional trajectory group is compared to the poorer group (ref).

**eTable 4** Odds ratio (OR) and risk difference for dementia by age groups where the poorer trajectory group are the reference, and E-values for each regression model are presented.

**eTable 1 Model fit of the multidimensional trajectories comparing two and three groups.**

| **Age group** |  | **Average posterior probability** | **Odds of correct classification** | **Traj (%)** | **n (%)** | **n (%) group assignment ≤ 0.7** | **BIC** |
| --- | --- | --- | --- | --- | --- | --- | --- |
| **>35 & ≤45** |  |  |  |  |  |  |  |
|  | **Two group model** |  |  |  |  | 397 (8.6) | -118220.0 |
|  | Group1 | .940 | 10.7 | 58.8 | 2739 (59.4) |  |  |
|  | Group2 | .925 | 18.0 | 41.2 | 1874 (40.6) |  |  |
|  | **Three group model** |  |  |  |  | 592 (12.8) | -117305.4 |
|  | Group1 | .895 | 19.0 | 31.5 | 1427 (30.9) |  |  |
|  | Group2 | .883 | 7.1 | 50.7 | 2385 (51.7) |  |  |
|  | Group3 | .898 | 42.1 | 17.9 | 801 (17.4) |  |  |
| **>45 & ≤55** |  |  |  |  |  |  |  |
|  | **Two group model** |  |  |  |  | 207 (8.8) | -61499.3 |
|  | Group1 | .930 | 12.7 | 51.4 | 1204 (51.3) |  |  |
|  | Group2 | .925 | 13.1 | 48.6 | 1143 (48.7) |  |  |
|  | **Three-Group model** |  |  |  |  | 373 (15.9) | -61121.0 |
|  | Group1 | .930 | 16.2 | 45.1 | 1056 (45.0) |  |  |
|  | Group2 | .856 | 14.2 | 29.1 | 693 (29.5) |  |  |
|  | Group3 | .839 | 15.3 | 25.8 | 598 (25.5) |  |  |
| **>55** |  |  |  |  |  |  |  |
|  | **Two group model** |  |  |  |  | 65 (10.3) | -16646.0 |
|  | Group1 | .917 | 12.2 | 47.6 | 301 (47.5) |  |  |
|  | Group2 | .922 | 10.7 | 52.4 | 333 (52.5) |  |  |
|  | **Three-Group model** |  |  |  |  | 79 (12.5) | -16612.4 |
|  | Group1 | .901 | 22.7 | 29.3 | 182 (28.7) |  |  |
|  | Group2 | .895 | 6.22 | 56.8 | 367 (57.9) |  |  |
|  | Group3 | .837 | 33.3 | 13.9 | 85 (13.4) |  |  |

*Note.* Traj Trajectory group, BIC Bayesian Information Criterion.

**eTable 2 Parameter estimates for the two multidimensional trajectories**.

| **Youngest-aged group,**  **>35 & ≤45** |  |  | **Better multidimensional trajectory** | | **Poorer multidimensional trajectory** | |
| --- | --- | --- | --- | --- | --- | --- |
|  |  |  |  | P-value |  | P-value |
|  | SBP | Intercept (SE) | 119.17 (0.28) | p<0.0001 | 133.87 (0.358) | p<0.0001 |
|  |  | Linear (SE) | 0.908 (0.061) | p<0.0001 | 1.937 (0.075) | p<0.0001 |
|  |  | Quadratic (SE) | -0.024 (0.003) | p<0.0001 | -0.058 (0.003) | p<0.0001 |
|  |  |  |  | |  | |
|  | DBP | Intercept (SE) | 77.05 (0.18) | p<0.0001 | 87.46 (0.23) | p<0.0001 |
|  |  | Linear (SE) | 0.188 (0.039) | p<0.0001 | 0.788 (0.047) | p<0.0001 |
|  |  | Quadratic (SE) | -0.019 (0.002) | p<0.0001 | -0.043 (0.002) | p<0.0001 |
|  |  |  |  | |  | |
|  | CRF | Intercept (SE) | 0.799 (0.015) | p<0.0001 | 0.336 (0.019) | p<0.0001 |
|  |  | Linear (SE) | 0.017 (0.003) | p<0.0001 | 0.014 (0.004) | p=0.0006 |
|  |  | Quadratic (SE) | -0.004 (<0.001) | p<0.0001 | -0.004 (<0.001 | p<0.0001 |
| **Middle- aged group,**  **>45 & ≤55** |  |  |  |  |  |  |
|  | SBP | Intercept (SE) | 120.66 (0.48) | p<0.0001 | 138.50 (0.51) | p<0.0001 |
|  |  | Linear (SE) | 1.025 (0.102) | p<0.0001 | 2.232 (0.105) | p<0.0001 |
|  |  | Quadratic (SE) | -0.024 (0.004) | p<0.0001 | -0.080 (0.005) | p<0.0001 |
|  |  |  |  | |  | |
|  | DBP | Intercept (SE) | 78.35 (0.291) | p<0.0001 | 89.72 (0.31) | p<0.0001 |
|  |  | Linear (SE) | 0.027 (0.061) | p=0.6598 | 0.473 (0.063) | p<0.0001 |
|  |  | Quadratic (SE) | -0.018 (0.003) | p<0.0001 | -0.042 (0.003) | p<0.0001 |
|  |  |  |  | |  | |
|  | CRF | Intercept (SE) | 0.848 (0.025) | p<0.0001 | 0.279 (0.025) | p<0.0001 |
|  |  | Linear (SE) | 0.013 (0.005) | p=0.0150 | 0.020 (0.005) | p=0.0003 |
|  |  | Quadratic (SE) | -0.003 (<0.001) | p<0.0001 | -0.004 (<0.001) | p<0.0001 |
| **Oldest-aged group, >55** |  |  |  |  |  |  |
|  | SBP | Intercept (SE) | 124.85 (1.11) | p<0.0001 | 145.65 (1.08) | p<0.0001 |
|  |  | Linear (SE) | 1.379 (0.231) | p<0.0001 | 2.913 (0.221) | p<0.0001 |
|  |  | Quadratic (SE) | -0.041 (0.010) | p<0.0001 | -0.120 (0.010) | p<0.0001 |
|  |  |  |  | |  | |
|  | DBP | Intercept (SE) | 79.62 (0.61) | p<0.0001 | 89.94 (0.59) | p<0.0001 |
|  |  | Linear (SE) | -0.223 (0.127) | p=0.0799 | 0.712 (0.123) | p<0.0001 |
|  |  | Quadratic (SE) | -0.013 (0.005) | p=0.0215 | -0.060 (0.005) | p<0.0001 |
|  |  |  |  | |  | |
|  | CRF | Intercept (SE) | 0.776 (0.048) | p<0.0001 | 0.347 (0.045) | p<0.0001 |
|  |  | Linear (SE) | 0.028 (0.011) | p=0.0093 | 0.017 (0.010) | p=0.1125 |
|  |  | Quadratic (SE) | -0.004 (<0.001) | p<0.0001 | -0.004 (<0.001) | p<0.0001 |

**eTable 3 Adjusted odds ratio (aOR) with 95% confidence interval (95% CI) for dementia stratified by age group and sex, where the better multidimensional trajectory group is compared to the poorer group (ref).**

| **Age group** | | **Men** | **Women** | **Both men and women** | **n** |
| --- | --- | --- | --- | --- | --- |
| *aOR (95% CI)* | | | | | |
| >35 & ≤45, | 0.77 (0.55, 1.07) | | 0.66 (0.46, 0.94) | 0.72 (0.56, 0.91) | 4613,  Women (52.5%) |
| >45 & ≤55, | 0.62 (0.44, 0.87) | | 0.64 (0.49, 0.84) | 0.63 (0.51, 0.78) | 2347,  Women (57.8%) |
| >55 | 0.46 (0.26, 0.79) | | 0.83 (0.55, 1.25) | 0.66 (0.48, 0.91) | 634,  Women (61.0%) |

**eTable 4 Odds ratio and risk difference for dementia by age groups where the poorer trajectory group are the reference, and E-values for each regression model are presented.**

| **Age groups** |  | **N** | **Odds ratio (95% CI)** | **Absolute risk difference (95% CI)** | **E-value point estimate (E-value CI)** |
| --- | --- | --- | --- | --- | --- |
| **>35 & ≤45** | Model 1* | 4613 | 0.67 (0.53, 0.84) | -0.02 (-0.04, -0.01) | 2.37 (1.65) |
|  | Model 2** | 4613 | 0.72 (0.56, 0.91) | -0.02 (-0.03, -0.01) | 2.14 (1.42) |
|  | Model 3*** | 4029 | 0.71 (0.54, 0.92) | -0.02 (-0.04, -0.00) | 2.19 (1.41) |
| **>45 & ≤55** | Model 1* | 2347 | 0.60 (0.49, 0.74) | -0.08 (-0.11, -0.05) | 1.89 (1.59) |
|  | Model 2** | 2347 | 0.63 (0.51, 0.78) | -0.07 (-0.10, -0.04) | 1.83 (1.52) |
|  | Model 3*** | 2091 | 0.57 (0.45, 0.71) | -0.09 (-0.12, -0.05) | 1.99 (1.65) |
| **>55** | Model 1* | 634 | 0.62 (0.45, 0.85) | -0.12 (-0.20, -0.04) | 1.86 (1.39) |
|  | Model 2** | 634 | 0.66 (0.48, 0.91) | -0.10 (-0.18, -0.02) | 1.76 (1.27) |
|  | Model 3*** | 553 | 0.70 (0.50, 1.00) | -0.08 (-0.17, -0.00) | 1.70 (1.03) |

*Note.* CI confidence interval.

*Unadjusted.

**Adjusted for sex, APOE ε4 status, education, marital status, and diabetes.

***Adjusted for sex, APOE ε4 status, education, marital status, diabetes, smoking, alcohol use frequency and anxiety- and depression-index.
